# Speciation on the Rocks: Integrated Systematics of the *Heteronotia spelea* Species Complex (Gekkota; Reptilia) from Western and Central Australia

**DOI:** 10.1371/journal.pone.0078110

**Published:** 2013-11-11

**Authors:** Mitzy Pepper, Paul Doughty, Matthew K. Fujita, Craig Moritz, J. Scott Keogh

**Affiliations:** 1 Division of Evolution, Ecology and Genetics, Research School of Biology, The Australian National University, Canberra, ACT, Australia; 2 Department of Terrestrial Zoology, Western Australian Museum, Perth, Western Australia, Australia; 3 Organismal and Evolutionary Biology, Harvard University, Cambridge, Massachusetts, United States of America; 4 Department of Biology, University of Texas at Arlington, Arlington, Texas, United States of America; Consiglio Nazionale delle Ricerche (CNR), Italy

## Abstract

The isolated uplands of the Australian arid zone are known to provide mesic refuges in an otherwise xeric landscape, and divergent lineages of largely arid zone taxa have persisted in these regions following the onset of Miocene aridification. Geckos of the genus *Heteronotia* are one such group, and have been the subject of many genetic studies, including *H. spelea*, a strongly banded form that occurs in the uplands of the Pilbara and Central Ranges regions of the Australian arid zone. Here we assess the systematics of these geckos based on detailed examination of morphological and genetic variation. The *H. spelea* species complex is a monophyletic lineage to the exclusion of the *H. binoei* and *H. planiceps* species complexes. Within the *H. spelea* complex, our previous studies based on mtDNA and nine nDNA loci found populations from the Central Ranges to be genetically divergent from Pilbara populations. Here we supplement our published molecular data with additional data gathered from central Australian samples. In the spirit of integrative species delimitation, we combine multi-locus, coalescent-based lineage delimitation with extensive morphological analyses to test species boundaries, and we describe the central populations as a new species, *H. fasciolatus* **sp. nov**. In addition, within the Pilbara there is strong genetic evidence for three lineages corresponding to northeastern (type), southern, and a large-bodied melanic population isolated in the northwest. Due to its genetic distinctiveness and extreme morphological divergence from all other *Heteronotia*, we describe the melanic form as a new species, *H. atra* **sp. nov.** The northeastern and southern Pilbara populations are morphologically indistinguishable with the exception of a morpho-type in the southeast that has a banding pattern resembling *H. planiceps* from the northern monsoonal tropics. Pending more extensive analyses, we therefore treat Pilbara *H. spelea* as a single species with phylogenetic structure and morphological heterogeneity.

## Introduction

There is growing consensus in the systematics community that best practice in species delimitation incorporates independent data from multiple sources [1]–[5]. In the current age of rampant species discovery, particularly in morphologically conservative groups [6]–[8], methods of delimiting species and testing species boundaries increasingly incorporate non-morphological characters, including chemical and auditory signals, ecology, geography, and molecular data [4], [9]. In particular, the acquisition of multi-locus genealogical data, along with advances in coalescent-based methods in the detection and description of species, are revolutionizing our ability to resolve problematic species complexes [10].

Australia has over 120 described gekkonid lizard species belonging to three endemic Gondwanan-age families; Carphodactylidae, Diplodactylidae, and Pygopodidae, and the more recently-arrived family Gekkonidae. Within the Gekkonidae, five genera occur in Australia, including *Christinus* along the southern continental margin [11], *Cyrtodactylus* and *Nactus* in the northern tropics, and *Heteronotia* and *Gehyra* widespread throughout the arid zone and tropics. The ancestors of these latter two taxa are thought to have originated in Asia, with colonization of Australia occurring in the mid and late Cenozoic, respectively [12], [13].

For many years *Heteronotia binoei* [14] and *H. spelea* [15] were the only members of the genus. Genetic work on *Heteronotia* and *Gehyra* began in the late 1970s when karyotyping revealed a complex of chromosome races [16]–[18] and the existence of parthenogenetic populations in *H. binoei* [19]. Although taxonomic progress followed with the species-rich *Gehyra*, the taxonomy of *Heteronotia* remained little changed, with only the description of *H. planiceps* [20] from the northern tropics. Consequently, only three species have been recognized within *Heteronotia* for decades [21]–[23].

More recently, *Gehyra* and *Heteronotia* have been the subject of multi-locus genetic studies which have unveiled further cryptic diversity, and shed light on evolutionary relationships and geographic distributions [12], [13], [24]–[26]. As in the 1970s, recent taxonomic progress has been made with *Gehyra* [24], [25], [27]–[29], but due to extremely high levels of cryptic diversity, particularly within the *H. binoei* and *H. planiceps* complexes, the taxonomy of *Heteronotia* has remained unchanged despite the increased understanding of genetic lineages within these species groups throughout Australia [12], [26].

Here we focus our phylogenetic study on populations currently referred to as *H. spelea*, occurring in the Pilbara region of Western Australia (type locality is Bamboo Creek, northern Pilbara), and those sometimes reported as *H. spelea* [30], *H. binoei* [23] or as *Heteronotia* sp. from the Central Ranges of the Northern Territory [31], [32]. In addition, records of *H. planiceps* also have been reported from the south-eastern Pilbara [20], [23]. Furthermore, a highly distinctive, large-bodied, melanic population was discovered on the Pilbara Biodiversity Survey in 2004 [33], [34], but has not been morphologically assessed in comparison to the other forms. Here we assess the systematics of these geckos based on detailed examination of morphological and genetic variation. We do not treat the *H. binoei* or *H. planiceps* species complexes here, other than to provide diagnoses that exclude them from *H. spelea* and allied taxa.

## Methods

### Molecular analyses

Our molecular data sets build on existing datasets from Pepper *et al*. [26] comprising the mitochondrial locus *nd2* and nine nuclear intron loci. This dataset included 15 representatives of *H. spelea* ‘central’, and 29 *H. spelea* from the Pilbara, including topotypic individuals. In addition, to resolve questions about geographic distributions, we collected new material for 17 *H. spelea* ‘central’ that we sequenced for *nd2*. These sequences have been deposited on Genbank (Accession numbers KF289018-KF289034). All field research and collecting of specimens was approved by the Australian National University Animal Experimentation Ethics Committee under the Animal Ethics Protocol Number F.BTZ.30.08, and the Northern Territory Parks & Wildlife Commission under the collecting permit 37093. We also include samples from the other *Heteronotia* species, including five *H. binoei* and eight *H. planiceps*, in each case spanning the known diversity. *Dixonius vietnamensis* (the sister taxon of *Heteronotia*) [35] was used as the outgroup to root the phylogenetic tree. For sample information and collection localities, see Table 1.

**Table 1:** Locality information for all individuals sampled in this study.

Lab ID	Museum	Museum #	Genus	Species	Latitude (dec.)	Longitude (dec.)	Location (nearest)
31241	SAM	ABTC31241	*Heteronotia*	*binoei*	−11.96670	141.90000	Mapoon Mission, Qld
31253	SAM	ABTC31253	*Heteronotia*	*binoei*	−15.70000	126.36667	Drysdale, WA
32437	SAM	ABTC32437	*Heteronotia*	*binoei*	−27.00000	133.31700	Chandler, SA
MKF118	MVZ	MKF118	*Heteronotia*	*binoei*	−27.95000	134.31300	Copper Hills, SA
MKF302	MVZ	MKF302	*Heteronotia*	*binoei*	−26.13190	113.40334	Useless Loop, WA
**Gko019**	**WAM**	**R96971**	***Heteronotia***	***planiceps***	**−15.71944**	**125.20694**	**Kings Cascade, WA**
Gko020	WAM	R106220	*Heteronotia*	*planiceps*	−16.10000	124.63333	Red Cone Hill, WA
Gko021	WAM	R113996	*Heteronotia*	*planiceps*	−15.35000	126.61667	Carson Escarpment, WA
Gko022	WAM	R132761	*Heteronotia*	*planiceps*	−15.29111	128.66917	Carlton Hill Station, WA
Gko025	WAM	R158015	*Heteronotia*	*planiceps*	−16.11417	123.69417	Koolan Island, WA
Gko026	WAM	R158024	*Heteronotia*	*planiceps*	−16.14583	123.74917	Koolan Island, WA
Gko027	WAM	R158033	*Heteronotia*	*planiceps*	−16.14722	123.77056	Koolan Island, WA
Gko254	SAM	ABTC29848	*Heteronotia*	*planiceps*	−15.76806	129.09000	Jarrnarm Escarpment, NT
Gko256	SAM	ABTC31418	*Heteronotia*	*fasciolatus* sp. nov.	−23.69611	134.03556	Undoolya Homestead, NT
Gko257	SAM	ABTC31432	*Heteronotia*	*fasciolatus* sp. nov.	−23.59000	134.47972	Ross River, NT
Gko258	SAM	ABTC31433	*Heteronotia*	*fasciolatus* sp. nov.	−23.69611	134.03556	Undoolya Homestead, NT
Gko259	SAM	ABTC31434	*Heteronotia*	*fasciolatus* sp. nov.	−23.59000	134.47972	Ross River, NT
Gko260	SAM	ABTC31435	*Heteronotia*	*fasciolatus* sp. nov.	−23.69611	134.03556	Undoolya Homestead, NT
Gko261	SAM	ABTC32871	*Heteronotia*	*fasciolatus* sp. nov.	−23.69611	134.03556	Undoolya Homestead, NT
Gko262	SAM	ABTC32988	*Heteronotia*	*fasciolatus* sp. nov.	−23.69611	134.03556	Undoolya Homestead, NT
Gko280	SAM	ABTC31199	*Heteronotia*	*fasciolatus* sp. nov.	−23.69611	134.03556	Undoolya Homestead, NT
Gko281	SAM	ABTC31436	*Heteronotia*	*fasciolatus* sp. nov.	−23.69611	134.03556	Undoolya Homestead, NT
Gko282	SAM	ABTC31437	*Heteronotia*	*fasciolatus* sp. nov.	−23.69611	134.03556	Undoolya Homestead, NT
Gko279	SAM	ABTC24118	*Heteronotia*	*fasciolatus* sp. nov.	−23.89000	133.71972	Mulga Dam, NT
Gko283	SAM	ABTC31636	*Heteronotia*	*fasciolatus* sp. nov.	−23.59000	134.47972	Ross River, NT
Gko284	SAM	ABTC31659	*Heteronotia*	*fasciolatus* sp. nov.	−23.59000	134.47972	Ross River, NT
Gko285	SAM	ABTC31726	*Heteronotia*	*fasciolatus* sp. nov.	−23.59000	134.47972	Ross River, NT
Gko286	SAM	ABTC31731	*Heteronotia*	*fasciolatus* sp. nov.	−23.59000	134.47972	Ross River, NT
MKF719	MCZ	-	*Heteronotia*	*fasciolatus* sp. nov.	−23.02284	134.93135	Hart's Range Racecourse, NT
MKF720	MCZ	-	*Heteronotia*	*fasciolatus* sp. nov.	−23.02284	134.93135	Hart's Range Racecourse, NT
MKF721	MCZ	-	*Heteronotia*	*fasciolatus* sp. nov.	−23.02284	134.93135	Hart's Range Racecourse, NT
MKF724	NTM	R36284	*Heteronotia*	*fasciolatus* sp. nov.	−23.02284	134.93135	Hart's Range Racecourse, NT
MKF725	MCZ	R188177	*Heteronotia*	*fasciolatus* sp. nov.	−23.02284	134.93135	Hart's Range Racecourse, NT
MKF728	NTM	R36312	*Heteronotia*	*fasciolatus* sp. nov.	−23.05736	134.94713	On roadside to Mt. Palmer, NT
MKF745	MCZ	-	*Heteronotia*	*fasciolatus* sp. nov.	−23.10062	134.53981	Cattlewater pass, NT
MKF746	MCZ	-	*Heteronotia*	*fasciolatus* sp. nov.	−23.10062	134.53981	Cattlewater pass, NT
MKF749	MCZ	R188178	*Heteronotia*	*fasciolatus* sp. nov.	−23.10062	134.53981	Cattlewater pass, NT
MKF750	MCZ	R188179	*Heteronotia*	*fasciolatus* sp. nov.	−23.10062	134.53981	Cattlewater pass, NT
MKF753	MCZ	-	*Heteronotia*	*fasciolatus* sp. nov.	−23.26703	134.32466	Arltunga tourist drive, NT
MKF754	NTM	R36319	*Heteronotia*	*fasciolatus* sp. nov.	−23.26703	134.32466	Arltunga tourist drive, NT
MKF755	MCZ	R188180	*Heteronotia*	*fasciolatus* sp. nov.	−23.26703	134.32466	Arltunga tourist drive, NT
MKF787	MCZ	R188182	*Heteronotia*	*fasciolatus* sp. nov.	−23.63799	132.73291	Ormiston Gorge, NT
MKF788	NTM	R36297	*Heteronotia*	*fasciolatus* sp. nov.	−23.63799	132.73291	Ormiston Gorge. NT
MKF789	MCZ	-	*Heteronotia*	*fasciolatus* sp. nov.	−23.62938	132.35660	Tylers pass, NT
MKF790	MCZ	-	*Heteronotia*	*fasciolatus* sp. nov.	−23.62938	132.35660	Tylers pass, NT
MKF791	MCZ	-	*Heteronotia*	*fasciolatus* sp. nov.	−23.62938	132.35660	Tylers pass, NT
MKF792	NTM	R36288	*Heteronotia*	*fasciolatus* sp. nov.	−23.62938	132.35660	Tylers pass, NT
MKF793	MCZ	R188183	*Heteronotia*	*fasciolatus* sp. nov.	−23.62938	132.35660	Tylers pass, NT
**Gko004**	**WAM**	**R110056**	***Heteronotia***	***atra*** ** sp. nov.**	**−21.03590**	**117.10658**	**5 km south of Lake Poongkaliyarra, WA**
**Gko005**	**WAM**	**R110075**	***Heteronotia***	***atra*** ** sp. nov.**	**−21.03590**	**117.10658**	**5 km south of Lake Poongkaliyarra, WA**
**Gko006**	**WAM**	**R110076**	***Heteronotia***	***atra*** ** sp. nov.**	**−21.03590**	**117.10658**	**5 km south of Lake Poongkaliyarra, WA**
**Gko443**	**WAM**	**R165222**	***Heteronotia***	***atra*** ** sp. nov.**	**−21.3413**	**117.1890**	**5 km south of Lake Poongkaliyarra, WA**
Gko442	WAM	R165152	*Heteronotia*	*spelea*	−23.0538	119.1770	PW3*, WA
Gko445	WAM	R170828	*Heteronotia*	*spelea*	−21.50580	119.41900	MBE06*, WA
Gko043	WAM	R145600	*Heteronotia*	*spelea*	−20.92860	118.67780	Port Hedland, WA
Gko446	WAM	R170892	*Heteronotia*	*spelea*	−21.5062	119.418	MBE1*, WA
Gko253	SAM	ABTC32937	*Heteronotia*	*spelea*	−23.37300	120.14200	Newman, WA
Gko255	SAM	ABTC11740	*Heteronotia*	*spelea*	−23.37300	120.14200	Newman, WA
Gko038	WAM	R132681	*Heteronotia*	*spelea*	−20.60028	120.29167	Shay Gap, WA
Gko440	WAM	R161285	*Heteronotia*	*spelea*	−21.3219	121.0020	NE09*, WA
Gko039	WAM	R135010	*Heteronotia*	*spelea*	−23.38611	119.62944	Mount Whaleback, WA
Gko438	WAM	R160085	*Heteronotia*	*spelea*	−21.3219	121.0020	NE09*, WA
Gko436	WAM	R111927	*Heteronotia*	*spelea*	−22.6098	120.7290	BDRN09*, WA
Gko437	WAM	R111986	*Heteronotia*	*spelea*	−22.6098	120.7290	BDRN09*, WA
Gko028	WAM	R97258	*Heteronotia*	*spelea*	−23.36667	120.13333	Wheelarra Hill, WA
Gko029	WAM	R102227	*Heteronotia*	*spelea*	−23.41139	115.89389	Barlee Range, WA
Gko030	WAM	R102380	*Heteronotia*	*spelea*	−23.40000	115.88333	Barlee Range, WA
Gko031	WAM	R102436	*Heteronotia*	*spelea*	−23.13056	115.99444	Goodeman Pool, WA
Gko032	WAM	R113545	*Heteronotia*	*spelea*	−22.83333	119.46667	Capricorn Roadhouse, WA
Gko033	WAM	R114561	*Heteronotia*	*spelea*	−23.08333	119.28333	Newman, WA
Gko034	WAM	R115832	*Heteronotia*	*spelea*	−23.11889	118.78278	The Governor, WA
Gko035	WAM	R115833	*Heteronotia*	*spelea*	−23.11889	118.78278	The Governor, WA
Gko036	WAM	R121394	*Heteronotia*	*spelea*	−22.86667	119.43333	Weeli Wolli, WA
Gko037	WAM	R132488	*Heteronotia*	*spelea*	−23.20000	117.66667	Paraburdoo, WA
Gko040	WAM	R135388	*Heteronotia*	*spelea*	−22.31056	117.32861	Mount Brockman Station, WA
Gko041	WAM	R135446	*Heteronotia*	*spelea*	−22.31056	117.32194	Mount Brockman Station, WA
Gko042	WAM	R135456	*Heteronotia*	*spelea*	−22.31056	117.32194	Mount Brockman Station, WA
Gko044	WAM	R157546	*Heteronotia*	*spelea*	−23.19417	118.81500	West Angeles, WA
Gko045	WAM	R157719	*Heteronotia*	*spelea*	−22.94083	118.90500	Newman, WA
**Gko278**	**SAM**	**ABTC11763**	***Heteronotia***	***spelea***	**−20.92000**	**120.20972**	**Bamboo Creek, WA**
**Gko288**	**SAM**	**ABTC32922**	***Heteronotia***	***spelea***	**−20.92000**	**120.20972**	**Bamboo Creek, WA**
**Gko287**	**SAM**	**ABTC32832**	***Heteronotia***	***spelea***	**−20.92000**	**120.20972**	**Bamboo Creek, WA**
Gko435	WAM	R111667	*Heteronotia*	*spelea*	−21.0364	117.1060	TCMBE*, WA
Gko439	WAM	R160145	*Heteronotia*	*spelea*	−21.3219	121.0020	NE09*, WA
Gko444	WAM	R170295	*Heteronotia*	*spelea*	−23.31810	117.87100	TCMBC05*, WA

**note:** Lab identification numbers (LabID) were given to each sample and used in the figures. Museum # refers to the voucher/tissue specimens held in the South Australian Museum (SAM), the Western Australian Museum (WAM), the Museum and Art Gallery of the Northern Territory (NTM), the Museum of Vertebrate Zoology, Berkeley (MVZ), and the Museum of Comparative Zoology, Harvard (MCZ). Locality information is given to the nearest named location as provided by the museums. Localities marked with “*” refer to sample sites from the Pilbara Biological Surveys (2004) & 2 (2005) (unpublished reports). Samples originating from type localities are indicated in **bold**.

For new material, DNA was extracted from RNA-Later preserved liver tissue using a standard salt extraction. PCR products were amplified and sequenced using primers and protocols described elsewhere [12], [26].

A maximum likelihood analysis of our complete *nd2* dataset, totaling 80 taxa, was conducted using RAxML-VI-HPC v7.0.4 [36]. The analysis implemented the general time-reversible substitution model with gamma-distributed rates among sites (GTR+G). Twenty runs with different starting trees were performed, and the most likely tree was chosen from this set. Support values were estimated from 1000 bootstrap replicates.

### Species tree inference

We used the hierarchical model implemented in *BEAST v. 1.6.0 [37], that co-estimates the species tree and all gene trees in one Bayesian MCMC analysis, to estimate a species tree phylogeny for the *H. spelea* complex. The *BEAST analysis requires *a priori* designation of species. To be conservative in our approach, we used the concatenated nDNA phylogeny as a heuristic to guide ‘candidate’ species for evaluation. We analyzed a reduced dataset that consisted of phased nuclear alleles for three individuals representing the diversity across each *H. spelea* population (Central, Melanic, Pilbara Northern, Pilbara Southern) as well as *H. planiceps*, resulting in 6 samples per population, and totaling 30 samples for 7853 base pairs of nDNA data. Models for each gene were selected under the BIC using PartitionFinder [38] (bzw1: HKY+I; dncl1: HKY; erh: HKY; frih: K80; lztfl1: HKY; nmes: HKY; rpl14: HKY; rpl35: K80+G; snrpd3: HKY). We unlinked loci and substitution models and used a Yule tree prior. Inspection of the frequency histograms using the diagnostic software Tracer v. 1.5 [39] of our initial analyses using a relaxed-clock model showed that the estimates of the coefficients of rate variation for all loci abutted against zero, meaning our data could not reject the use of a strict clock [40]. To reduce the number of parameters in the analysis and to improve precision [41] we used a strict clock model for final analyses. For the mean rate priors for the strict clock model, we specified a normal distribution with a lower bound of 1e-3 and with an upper bound of 1. We conducted four separate runs, with samples drawn every 10,000 steps over a total of 100,000,000 steps, with the first 10% discarded as burn-in. Acceptable convergence to the stationary distribution was checked by inspecting the posterior samples using the diagnostic software Tracer v1.5 [39]. Effective sample sizes were >200 for all parameters. All runs produced the same topology with very similar posterior probabilities, so we combined runs to generate a single consensus tree.

### Bayesian species delimitation

We used a Bayesian modeling approach to calculate posterior probabilities of putative species delimitations within populations of Pilbara *H. spelea*.

We used the program Bayesian Phylogenetics and Phylogeography (BPP v. 2.2) [42], [43], which accommodates the species phylogeny as well as lineage sorting due to ancestral polymorphism. We used the same dataset as for the species tree analysis, with our guide tree topology specified using the relationships inferred from *BEAST ((((*H. spelea* ‘southern’, *H. spelea* ‘northeastern’), *H. spelea* ‘melanic), *H. spelea* ‘central’), *H. planiceps*).

Following the method of Leache & Fujita [44], we initially used three different combinations of prior distributions for the ancestral population size (*θ*) and root age (*τ*), with both priors assigned a gamma G(*α*, *β*) distribution, with a prior mean = *α*/*β* and prior variance = *α*/*β* ^2^. (1) a relatively large ancestral population with deep divergences (*θ* = 1, 10; *τ* = 1, 10), both with a prior mean = 0.1 and variance = 0.01, (2) a relatively small ancestral population and shallow divergences (*θ* = 2, 2000; *τ* = 2, 2000), both with a prior mean = 0.001 and variance = 5×10^−7^, and (3). a relatively large ancestral population with shallow divergences (*θ* = 1, 10; *τ* = 2, 2000). In addition, to evaluate the effect of the prior distributions on posterior probabilities, we performed another analysis where the prior distributions for *θ* and *τ* were estimated directly from our dataset (*θ* = 4, 100, with a prior mean = 0.04 and variance = 4×10^−4^; *τ* = 7, 40, with a prior mean = 0.175 and variance = 0.004). For *θ*, we calculated average pairwise distance (Dxy) in MEGA [45] for each putative “species” to obtain an average Dxy (0.0345) and then we fitted this to a Gamma distribution in R v. 2.15.0 [46]. For *τ* we used our concatenated data to estimate the phylogeny using BEAST v. 1.7.5 [47] in order to obtain the root age (0.214) which also was fitted to a Gamma distribution. The other divergence time parameters were assigned the Dirichlet prior [42]. Each analysis was run at least twice to confirm consistency between runs. In addition, to test the informativeness of our data, we also ran the analyses without data.

### Morphological analysis

Following from a conservative interpretation of the genetic data (see below), we used four groups for the morphological analysis (‘central’, ‘melanic’, north-eastern ‘*sensu stricto*’ [*s.s.*], and ‘southern’). We examined specimens from the collections of the Western Australian Museum (WAM), Northern Territory Museum (NTM), and the Harvard Museum of Comparative Zoology (MCZ), where type material is deposited. For *H. spelea* ‘melanic’ (n = 6), this represents all the material available in collections. Most specimens examined were genotyped and could be assigned to a group based on their genetic clade. Where specimens were not genotyped, they were only assigned to a group if their locality unambiguously fell in the known geographic range of a group. In addition, non-genotyped individuals of *H. spelea* ‘melanic’ have a highly distinctive morphology compared to all other *Heteronotia*.

The following measurements were taken with electronic calipers to the nearest 0.1 mm (see Table S1 for more detailed summaries of characters measured): TailW - tail width; TrunkL - trunk length; ArmL - arm length; LegL - leg length; HeadL - head length; EarSnout - ear to snout distance; HeadD - head depth; HeadW - head width; IntOrb - inter orbital distance. Snout-vent length (SVL) and tail length (TailL) of original tails were measured with a rule to the nearest 1 mm. Fine-scale measurements of EarL - ear length and OrbL - orbit length were made using a microscope eyepiece. Scale counts were carried out for NarScales - number of narial scales, Supralab - number of supralabial scales, InfraLab - number of infralabial scales, FingerLam - number of subdigital lamellae on fourth finger, and ToeLam - number of subdigital lamellae on fourth toe. Scale counts and external observations were made using a dissecting microscope. Measurements and scale counts based on right side of animals. Individuals were scored for sex by the presence of inverted hemipenes, pre-cloacal pores, eggs, or by dissection.

In total 21 characters were evaluated in 57 specimens, but not all characters could be measured in all specimens. After first examining all continuous body size measurements for the variation, we chose nine for further multivariate analyses (see Table 2), and excluded tail length, tail width, head depth and inter-orbital distance. We used Principal Components Analysis (PCA), which does not identify groups *a priori*, and Discriminant Function Analysis (DFA), where the groups were specified *a priori*, to examine the patterns of relationship and discriminating power of the nine body proportion characters (natural log transformed) with the statistics software JMP 8.0. Because sample sizes were small, and we could find no evidence of sexual dimorphism in the body proportion variables, we pooled males and females. The first PC was interpreted as representing variation in body size and the second PC summarized shape differences. We then performed DFA on the ln-transformed data to examine if body shape differences would be sufficient to distinguish species when they were specified *a priori*. We first performed DFA on the nine body proportion variables and then did stepwise removal of variables from the model, based on F ratios, to examine the influence on DFA performance.

**Table 2:** Summaries of characters and ratios measured for members of the *H. spelea* species complex.

Character	*H. spelea (n = 28)*	*H. atra sp. nov. (n = 6)*	*H. fasciolatus* sp. nov (n = 15)
**SVL**	49.70±0.89	58.50±1.92	47.67±1.21
	(42.00–55.50)	(54.00–62.50)	(34.00–57.00)
**TrunkL**	20.85±0.52	25.29±1.12	20.80±0.71
	(16.6–26.52)	(23.01–27.72)	(14.48–25.18)
**HeadL**	14.34±0.25	16.44±0.55	13.66±0.35
	(11.83–16.19)	(14.78–17.27)	(9.78–16.33)
**HeadW**	9.81±0.22	10.80±0.47	10.32±0.30
	(8.08–11.28)	(9.48–11.72)	(7.01–13.17)
**ArmL**	7.11±0.13	8.56±0.27	6.59±0.17
	(6.10–8.33)	(7.50–9.55)	(5.13–7.65)
**LegL**	8.84±0.16	10.71±0.35	8.30±0.22
	(7.75–10.34)	(9.59–11.51)	(5.92–9.80)
**OrbL**	1.63±0.03	1.84±0.06	1.45±0.04
	(1.28–1.8)	(1.68–2.00)	(1.00–1.68)
**EarL**	0.53±0.02	1.07±0.05	0.53±0.03
	(0.32–0.64)	(0.72–1.2)	(0.36–0.72)
**EarSnout**	13.35±0.23	15.09±0.49	12.62±0.31
	(10.94–14.92)	(13.95–15.59)	(8.96–14.76)
**NarScales**	3–96%	3–83%	3–100%
	4-4%	4–17%	-
**SupraLab**	7-4%	8–17%	7-7%
	8-7%	9–17%	8–53%
	9–50%	10–67%	9–33%
	10–39%		10-7%
**InfraLab**	6-4%	7–17%	5–7%
	7–64%	8–83%	6–40%
	8–29%	-	7–53%
	9-4%	-	-

See Table S1 for abbreviations of characters measured.

Mean±SD (range).

### Nomenclatural Acts

The electronic edition of this article conforms to the requirements of the amended International Code of Zoological Nomenclature, and hence the new names contained herein are available under that Code from the electronic edition of this article. This published work and the nomenclatural acts it contains have been registered in ZooBank, the online registration system for the ICZN. The ZooBank LSIDs (Life Science Identifiers) can be resolved and the associated information viewed through any standard web browser by appending the LSID to the prefix “http://zoobank.org/”. The LSID for this publication is: urn:lsid:zoobank.org:pub:39D62054-632B-4F01-A960-504D24773CA2. The electronic edition of this work was published in a journal with an ISSN, and has been archived and is available from the following digital repositories: PubMed Central, LOCKSS.

## Results

### Molecular genetics

**Figure 1:**
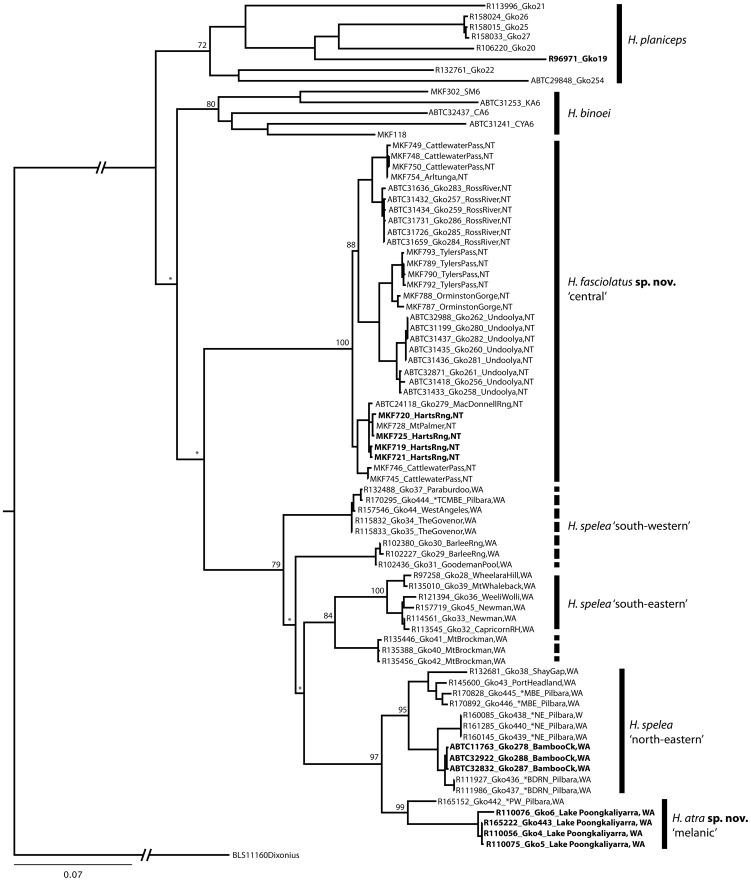
Maximum likelihood phylogram of *Heteronotia* species based on 80 mtDNA *nd2* sequences. The core material is based from the study of Pepper et al. (2011), and here we have included an additional 17 samples of the central Australian population, *H. fasciolatus* **sp. nov.** Samples originating from type localities are indicated in **bold**. (A “*” indicates bootstrap support <50.)

Here we present a Maximum Likelihood mtDNA phylogeny of all the specimens used in our morphological analysis (Fig. 1). Our *nd2* phylogeny inferred the same overall topology as that of Pepper *et al*. [26]. The additional samples of *H. spelea* ‘central’ uncovered a number of weakly diverged clades not present in the previous study, increasing the maximum mtDNA uncorrected ‘*P*’ genetic distance within this taxon from 0.0365 to 0.0403. It is clear that samples from the Central Ranges are genetically divergent in both mtDNA (average *P* distance = 0.1340) and nDNA (average *P* distance = 0.0129) from Pilbara *H. spelea*.

**Figure 2:**
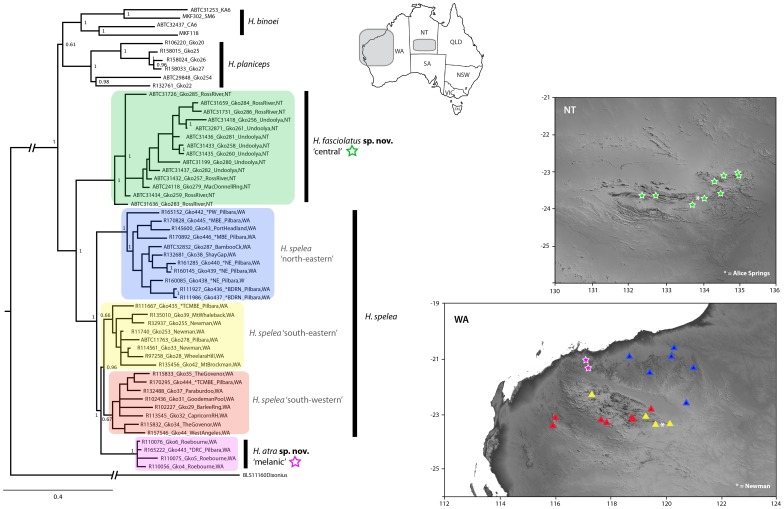
Genetic lineages of the *H. spelea* complex and their geographic distributions. Representative phylogram based on Bayesian analysis of nine nDNA loci of the *Heteronotia spelea* complex plus outgroups from Pepper *et al.* (2011). Values refer to Bayesian posterior probabilities. The outline of Australia shows the distribution of the Pilbara and central Australian lineages, while the insets show the detailed topography of these regions with sample localities colored to match the phylogeny. The distribution of *H. fasciolatus* **sp. nov.** and *H. atra* **sp. nov.** are indicated with green and pink stars, respectively.

Within Pilbara *H. spelea*, the mtDNA analysis found a number of divergent lineages, with largely unresolved relationships among them (Fig. 1). *Heteronotia spelea* ‘s.s.’ from the north-eastern Pilbara, and *H. spelea* ‘melanic’ from the north-west Pilbara form a well-supported clade (97/100 bootstrap support), and individuals of *H. spelea* ‘southern 1’ (south-eastern) also form a clade. However, the remaining *H. spelea* ‘southern 2’ (south-western) are paraphyletic in our mtDNA phylogeny, with short internal branch lengths. The previous study of Pepper *et al*. [26] based on nine nuclear loci (and not including mtDNA) recovered three well-supported lineages: north-eastern (*H. spelea* ‘s.s.’), north-western (*H. spelea* ‘melanic’), and southern, the latter of which includes both *H. spelea* ‘south-western’ and *H. spelea* ‘south-eastern’ which are characterized by shallow branch-lengths and poor support on internal nodes. (see Fig. 2).

### Species tree inference

**Figure 3:**
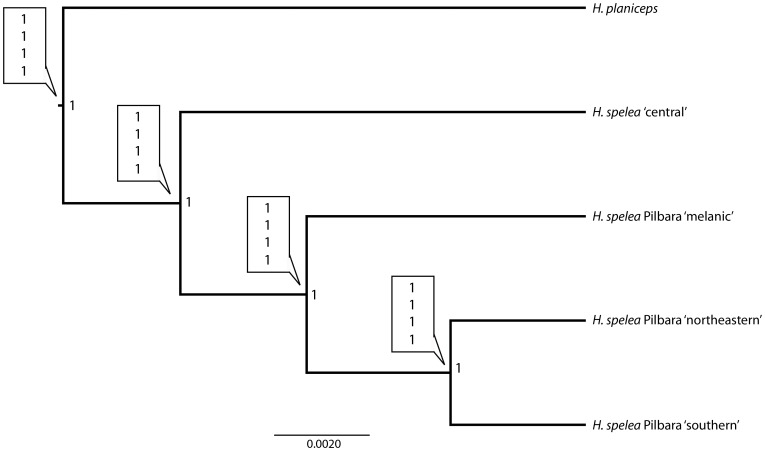
Species tree estimation and Bayesian species delimitation results. Each node of the tree is labeled with the posterior probability obtained from the *BEAST analysis (outside the box) and the posterior probabilities of the species split estimated using the rjMCMC under different combinations of prior distributions of *θ* and *τ* (inside the box) in the order: 1 – prior means = 0.1; 2 – prior means = 0.001; 3 – prior mean *θ* = 0.1, prior mean *τ* = 0.001; 4 – prior mean *θ* = 0.04, prior mean *τ* = 0.175.

The species tree resulting from the *BEAST analysis of the four *H. spelea* populations (central, melanic, northeastern, southern) resolves the relationships between these lineages with strong support (Fig. 3). *Heteronotia spelea* ‘central’ is inferred as sister to the three Pilbara populations (posterior probability = 1), and unlike either the mtDNA or the concatenated nDNA results, the *BEAST analysis supports a topology where *H. spelea* ‘northeastern’ and ‘southern’ group together, and are sister to *H. spelea* ‘melanic’.

### Bayesian species delimitation

The Bayesian species delimitation results for *H. spelea* are presented in Fig. 3. All species in the guide tree are well supported when assuming three species in the Pilbara (melanic, Pilbara northeastern, Pilbara southern) and another in central Australia (central). This result is not changed when mtDNA data is excluded from the analysis, or with the different prior distributions for *θ* and *τ*. We are confident that BPP analyses are performing reasonably, as when run without data, there was no support for species in the guide tree.

### Morphological analyses

Table 2 summarizes the morphological data for both continuous and discrete variables. Preliminary PCA analyses of the nine continuous characters demonstrated that the two genetic groups of non-melanic Pilbara *H. spelea* (‘northeastern’ and ‘southern’) were morphologically homogeneous with regard to shape but divergent from *H. spelea* ‘melanic’ (data not shown). Therefore, we pooled the Pilbara *H. spelea* ‘northeastern’ and ‘southern’ samples for further shape comparisons against the *H. spelea* ‘central’ and *H. spelea* ‘melanic’ clades based on the nine continuous characters. Our final morphometric dataset included six individuals of *H. spelea* ‘melanic’, 15 *H. spelea* ‘central’, and 28 non-melanic Pilbara *H. spelea*.

**Figure 4:**
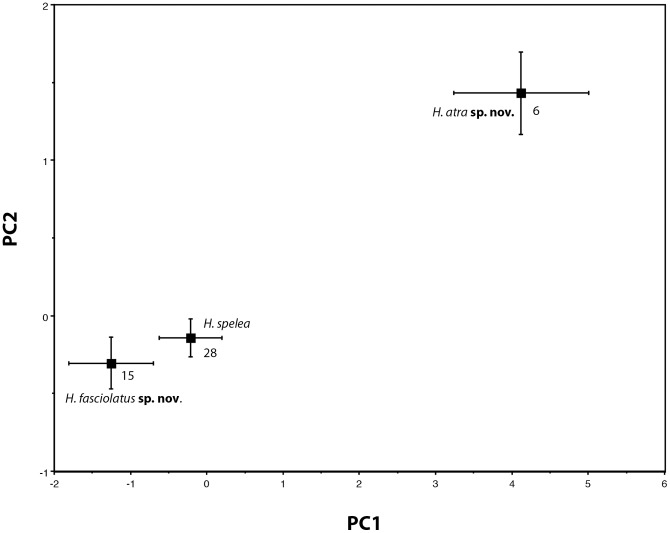
Summary of results for the PCAs of the morphological data for 49 *Heteronotia* specimens. Mean PC scores and standard deviations are shown with sample sizes noted.

PCA analyses demonstrate that *H. spelea* ‘central’ and non-melanic Pilbara *H. spelea* are morphologically similar to each other in size and shape, and that *H. spelea* ‘melanic’ is morphologically divergent from the other two in size and shape. We summarize the results of our PCA analysis on the nine continuously distributed characters in Fig. 4 where we show mean PC scores and standard deviations. PC1 explains 79.1% of the variation, PC2 explains 7.7% of the variation and the mean PC scores varied significantly among the three groups (PC1: F _2,46_ = 13.5, *P*<0.0001; PC2: F _2,46_ = 17.2, *P*<0.0001) but not between *H. spelea* ‘central’ and non-melanic Pilbara *H. spelea*. As expected, PC1 was very highly correlated with SVL (SVL, r^2^ = 0.97, *P*<0.001) and PC2 summarized shape differences among the species, with the highest loadings on head width and ear length. Additional PCs explained negligible amounts of the variation. DFA, in which group identity was specified *a priori* and based on the nine continuous characters was able to correctly identify 94% of the specimens to group based on body proportions alone. DFA correctly identified 100% of the *H. spelea* ‘melanic’, 93.3% of *H. spelea* ‘central’ and 92.8% of non-melanic Pilbara *H. spelea*. This high level of correct identification persisted following removal of up to four of the nine continuous characters.

**Figure 5:**
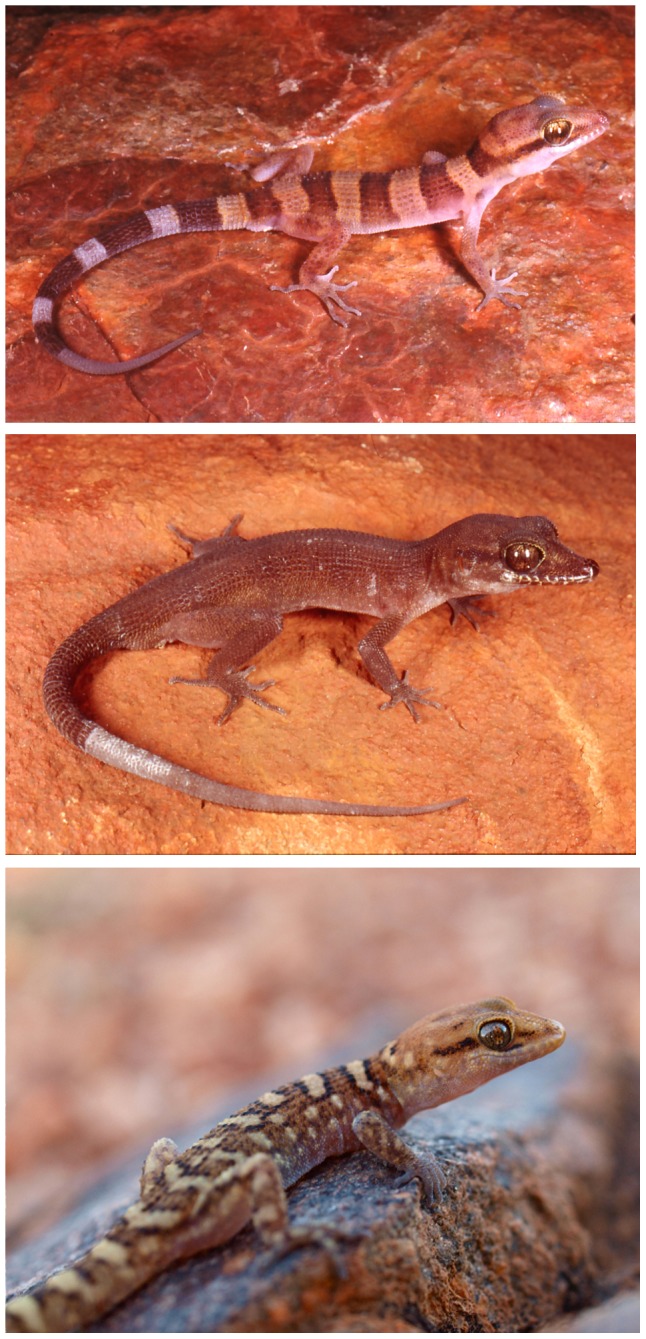
Photographs in life. (A) *Heteronotia spelea* in life: Paraburdoo, WA (image: B. Maryan); (B) *Heteronotia atra* **sp. nov.** in life: 5 km south of Lake Poongkaliyarra, WA (image: B. Maryan); (C) *Heteronotia fasciolatus* **sp. nov.** in life: Harts Range, central Australia, NT (image: M. Pepper).

**Figure 6:**
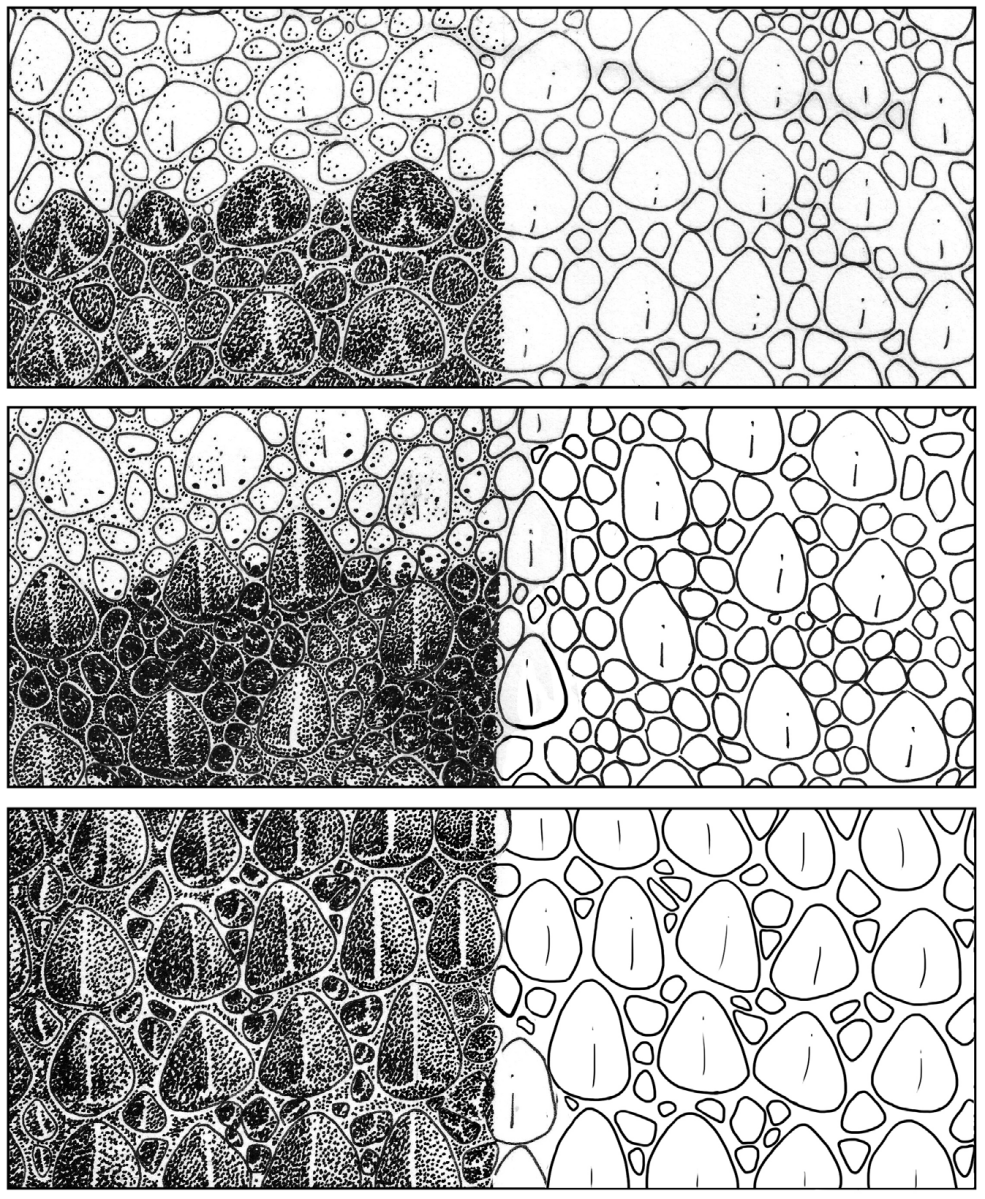
Arrangement of enlarged dorsal tubercles and small granule interspace scales in species of the *Heteronotia spelea* complex. (A) Pilbara *H. spelea*, (B) *H. fasciolatus* **sp. nov.**, (C) *H. atra* **sp. nov.**

While non-melanic Pilbara *H. spelea* and *H. spelea* ‘central’ were morphologically homogeneous based on size and shape (Fig. 4), they differed in several discrete characters. Within Pilbara *H. spelea*, three morphotypes are apparent. Specimens from the type population in the north-eastern Pilbara have four strongly-contrasting dorsal bands with straight edges (Fig. 5a). Most of the southern Pilbara individuals also share this pattern, with the exception of several individuals from the south-eastern Pilbara near Newman, that differ in having five or six bands. The occipital band of these individuals is in contact with the temporal stripe behind the eye, similar to *H. planiceps* [20], [21]. The enlarged dorsal tubercles of north-eastern, south-eastern, and south-western Pilbara *H. spelea* are moderately spaced, usually with at least one smaller granule separating them (Fig. 6a). Individuals from the north-western *H. spelea* ‘melanic’ Pilbara population have a highly distinct morphotype characterized by a large body size, large ear opening, and dark brown/black coloration (Fig. 5b). The enlarged dorsal tubercles are densely spaced and generally in contact posterior and anterior to the scale, with few (at most one) smaller granules separating them on either side (Fig. 6c).

**Figure 7:**
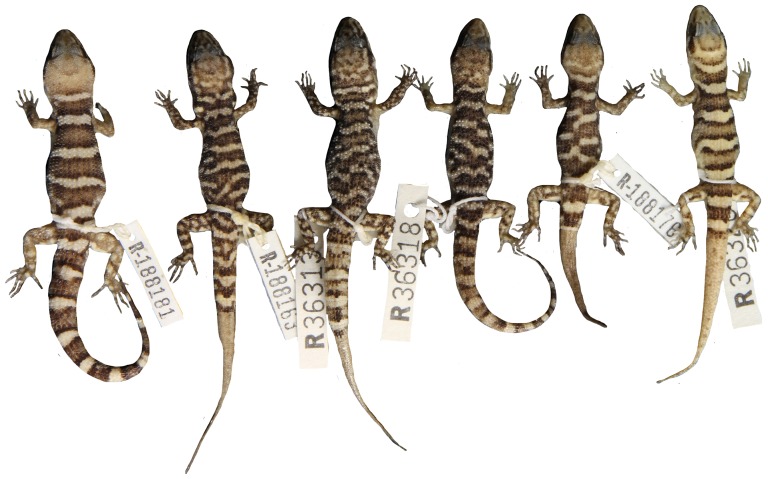
Variation of dorsal pattern within sympatric *Heteronotia binoei* in central Australia.

**Figure 8:**
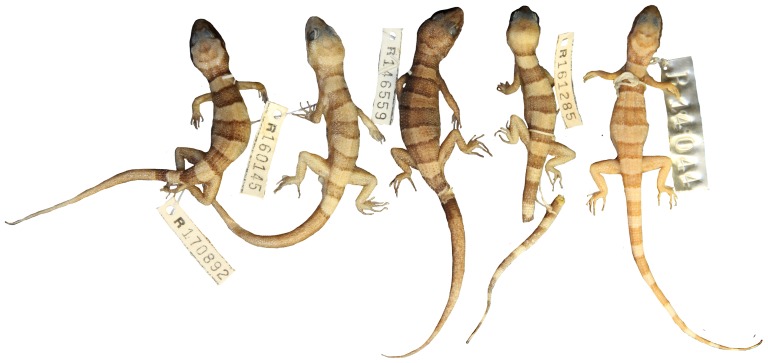
Variation of dorsal pattern within *Heteronotia spelea* s.s. (north-eastern).

**Figure 9:**
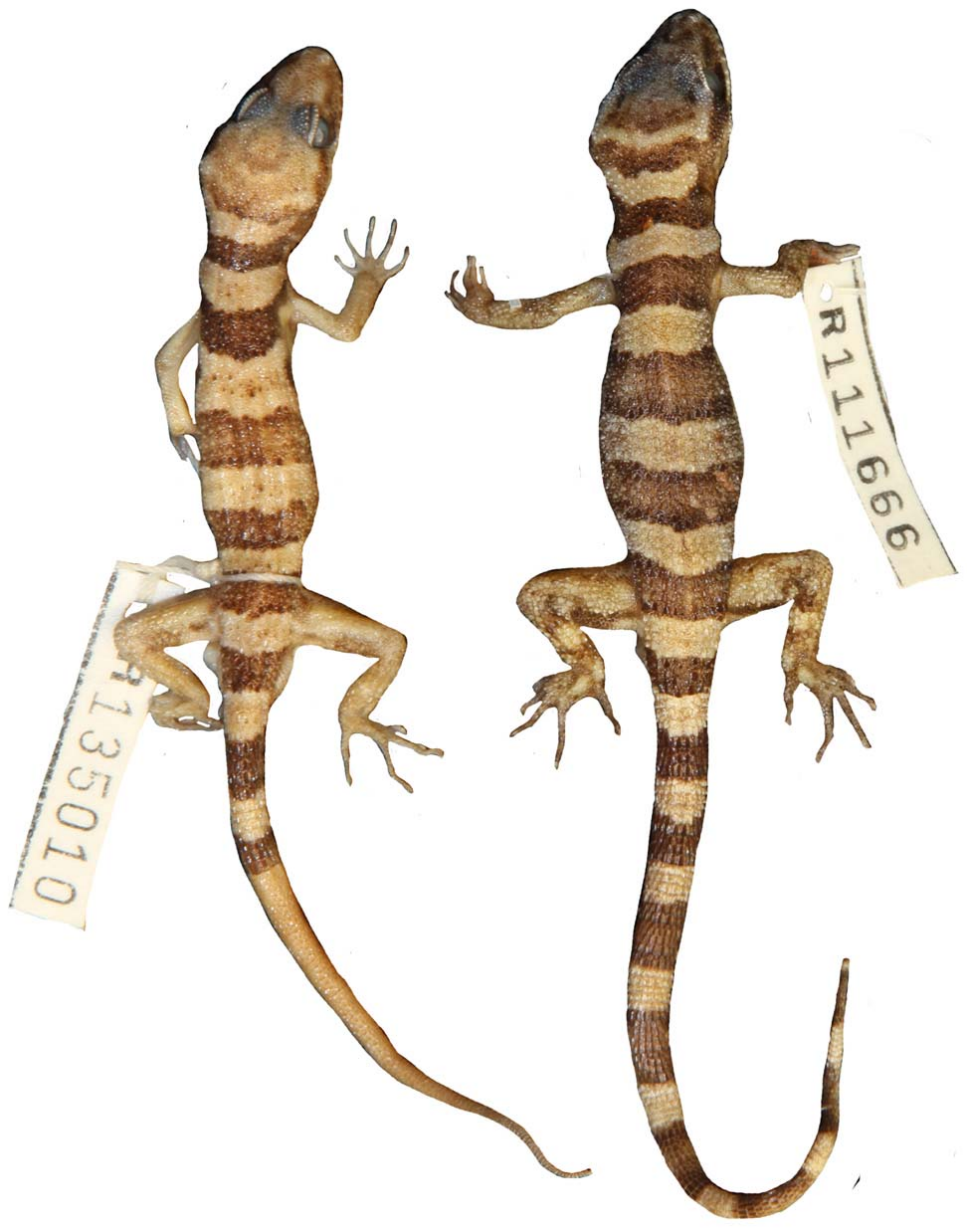
Dorsal pattern within *Heteronotia spelea* ‘south-eastern’. Note the occipital band of these individuals is in contact with the temporal stripe behind the eye, giving the appearance of an extra dorsal band (5 instead of 4).

**Figure 10:**
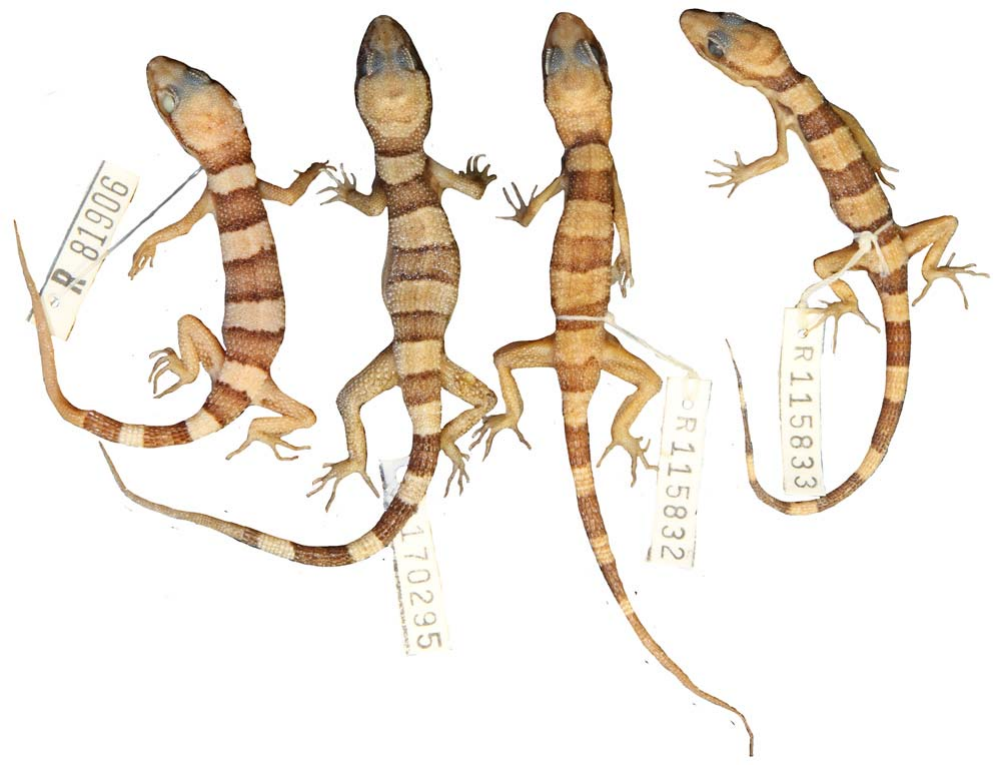
Variation of dorsal pattern within *Heteronotia spelea* ‘south-western’.

**Figure 11:**
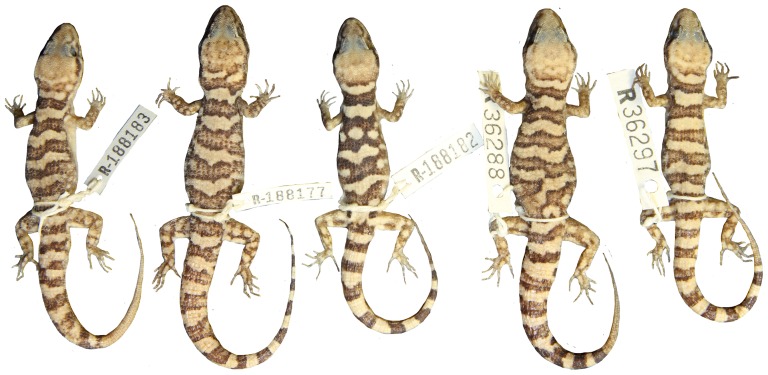
Variation of dorsal pattern within *Heteronotia fasciolatus* sp. nov.

**Figure 12:**
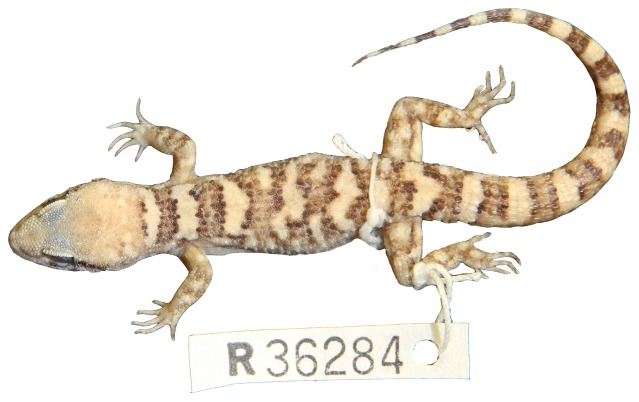
*Heteronotia fasciolatus* sp. nov. (holotype, NTM R36284).

**Figure 13:**
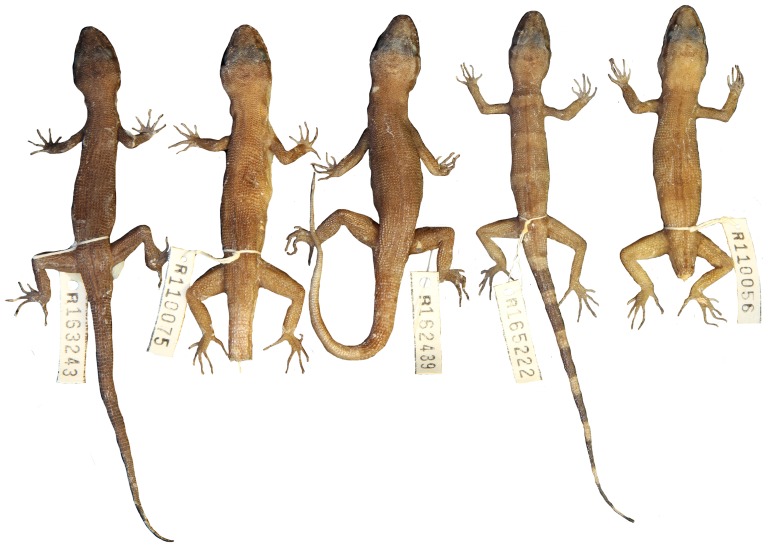
Variation of dorsal pattern within *Heteronotia atra* sp. nov.

**Figure 14:**
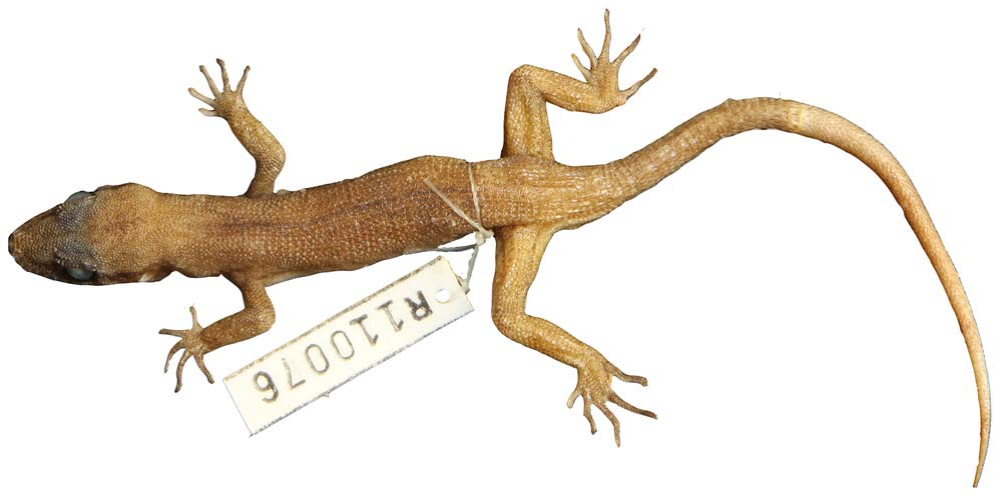
*Heteronotia atra* sp. nov. (holotype, WAM R110076).

*Heteronotia spelea* ‘central’ is distinctive in a number of morphological characters compared to Pilbara *H. spelea*. Specimens from populations in the Central Ranges have a broad, pale-colored head, with numerous (five to eight) strongly-contrasting dorsal bands with irregular edges (Fig. 5c). The enlarged and keeled dorsal tubercles are the most widely spaced in this lineage, typically with at least two smaller granules between them (Fig. 6b). Sympatric *H. binoei* of the chromosome lineage CA6 share a similar banding pattern, and can sometimes appear morphologically indistinguishable by eye (Fig. 7). For photographs showing variation of dorsal pattern within all *H. spelea* linages, see Figs. 8–14.

## Discussion

### Taxonomic conclusions

Currently recognized *Heteronotia* species are characterized by high morphological variation within, and low morphological variation between species. In this context, the integration of molecular and phenotypic evidence in species delimitation is especially important [4]. Recent genetic studies using multiple loci have identified substantial cryptic diversity within both the *H. planiceps* and *H. binoei* species complexes [12], [26], and preliminary morphological assessments of these groups suggest resolving the taxonomy will be challenging. In contrast, the *H. spelea* complex is characterized by considerably less genetic variation, and there are a number of discrete morphological characters that, in conjunction with geographic locality, reliably can be used to discriminate between species.

*Heteronotia spelea* ‘central’ is geographically limited to the Macdonnell Range mountain system in central Australia, as well as a number of smaller adjacent ranges to the north-east. The genetic distinctiveness of this taxon in both mtDNA and nDNA is well established [26]. In addition, a cytogenetic study found individuals of *H*. *spelea* ‘central’ (referred to as *H. sp.*) to be unique from other *Heteronotia* (including Pilbara *H. spelea*) in the placement of the nucleolar organizing region on the short arms of one of the chromosomes [31]. Furthermore, these individuals also differed from the sympatric *H. binoei* CA6 chromosome race by having no interstitial C-bands [31]. Stewart [32] informally proposed the name *H. fasciolatus* on the examination of four karyotyped specimens from Undoolya Station in Central Australia. However, this does not constitute a valid species description under the ICZN, and there is no mention of this name anywhere in the published literature. On the basis of the accumulation of genetic evidence, and in conjunction with its largely distinctive external morphology and isolated distribution, here we formally describe populations of *H. spelea* ‘central’ as new species, *H. fasciolatus* **sp. nov.**

Three geographically isolated and genetically distinctive clades occupy the Pilbara region. The type locality of *Heteronotia spelea* is Bamboo Creek in the north-eastern Pilbara. While morphologically indistinguishable from most *H. spelea* in the southern Pilbara (with the exception of the 5-banded morphotype near Newman), this northern lineage is distinct for nDNA [26] but is nested within *H. spelea* ‘southern’ for mtDNA. In addition, the phylogenetic structure between populations in the southern Pilbara also is incongruent between phylogenies based on mtDNA and those based on the concatenated nDNA, with analyses of the nDNA data showing relationships among the southern-distributed populations are poorly resolved with short branch lengths between clades (Fig. 2). Given the incongruence and short branches, we consider the boundaries and phylogenetic relationships among these southern clades as unresolved based on our data but note that future work with dense sampling of populations and many more loci may resolve these relationships. We found substantial incongruence between topologies derived from our mtDNA analysis, the concatenated nDNA analysis, and our species tree analysis. *Heteronotia spelea* Pilbara ‘southern’ is paraphyletic in the mtDNA phylogeny, with some individuals more closely related to *H. spelea* Pilbara ‘northeastern’ and *H. spelea* ‘melanic’. The concatenated nDNA analysis infers *H. spelea* Pilbara ‘southern’ to be most closely related to *H. spelea* ‘melanic’, while the species tree analysis groups *H. spelea* ‘southern’ and ‘northeastern’ together to the exclusion of *H. spelea* ‘melanic’. Given that species-tree methods provide a more realistic estimate of relationships than concatenated approaches [37], and in addition to morphological similarities, we consider *H. spelea* ‘northeastern’ and ‘southern’ as a single species with phylogenetic structure, pending further analyses using more loci, and additional sampling from around contact zones.

*Heteronotia spelea* ‘melanic’ is the most morphologically distinctive of all *Heteronotia* species, with its melanic phenotype and greatly enlarged ear opening. Individuals of this taxon form a monophyletic group at the end of a long branch in both the mtDNA and nDNA phylogenies, clustering with southern *H. spelea* for nDNA and northern *H. spelea* for mtDNA. Together with large differences in overall body shape and scalation, we describe this population as new species, *H. atra* **sp. nov.** *Heteronotia atra* **sp. nov.** is only known from two localities in the north-west Pilbara - five samples were collected from the top of a massive, heavily weathered dolerite mesa near Harding Dam in the north-west Pilbara, while another individual was found 30 km away in Millstream National Park. The habitat on the mesa is described as a bare, black boulder scree with many stony crevices and scattered shrubs. Color variation in reptiles, including melanism, is known to coincide with differences in substrate color [48], [49]. For example, populations of desert-horned lizards and common side-blotched lizards have melanic forms on lava flows in southern California [48], while some fence lizards have melanic populations on dark lava flows in southern New Mexico [50]. In some snakes, melanism is hypothesized to be a thermoregulatory adaptation to cool environments, such as island populations of the common garter snake [51] and adders in southwest Sweden [52]. The unique habitat associated with the heavily weathered, dark dolerites of the mesa, as well as the lack of vegetation cover, may play an important role in the evolution of melanism in *H. atra* **sp. nov**. Further work, including understanding the thermal tolerances of this species compared to other *Heteronotia* geckos, predator/prey interactions in this unique habitat, as well as the molecular basis for the observed color variation [47] will shed further light on this population of melanic geckos.

### Phylogeographic comments

The three main clades of Pilbara *H. spelea* recovered in the nDNA phylogeny of Pepper *et al.* [26] have non-overlapping distributions within the Pilbara (Fig. 2). The northern lineage, type *H. spelea*, occupies the undulating granitic hills of the north-eastern portion of the Pilbara geological craton. A second lineage *H. spelea* ‘southern’ is distributed throughout the uplands of the Hamersley plateau in the southern Pilbara, and in adjacent ranges just outside the southern craton margin. This group is separated from northern Pilbara populations by the Fortescue River valley and marshes, where saxicolous lizards do not occur. A third lineage *H. atra* **sp. nov**. is only known from an isolated locality of dolerite mesas near Karratha in the north-west Pilbara. These phylogeographic patterns (southern, north-eastern and north-western) have been identified in a number of other Pilbara reptiles [53] including geckos [54], [55], pebble-mimic dragons [56], spiny-tailed skinks [57] and *Ctenotus* skinks (D. Rabosky, P. Doughty, unpublished data), and are likely related to differences in underlying geological substrate and associated topography, as well as drainage divides across the Pilbara craton [53].

The distribution of *H. fasciolatus* **sp. nov.** is limited to the Macdonnell Ranges of central Australia including the Harts Range to the north and east. This species is not found in adjacent southern mountain systems such as the James or Petermann ranges, where strongly banded individuals, some resembling *H. fasciolatus*, are genetically determined to be *H. binoei* (Moritz *et al.* unpublished). The mountain ranges of central Australia are major topographic features of Australia's central arid zone that are completely isolated by surrounding sand deserts. It has been postulated that while coastal mountain ranges such as in the Pilbara or Kimberley would have been wetter and more thermally buffered during past periods of peak aridification [58], the uplands in central Australia may have experienced more severe arid conditions, including temperatures that were much colder than at present [59]. This hyper-arid climatic history is thought to have repeatedly extinguished narrowly endemic taxa in central Australia during the Pleistocene [60]. A similar pattern was identified by Linder [61] of plant endemism in sub-Saharan Africa, where the expansion of deserts during arid cycles promoted a series of extinctions, even in apparently suitable mountain refugia. The low level of genetic diversity within *H. fasciolatus* **sp. nov.** in central Australia compared to other *Heteronotia* lineages in rocky ranges of the Pilbara, Kimberley and Top End [26] provides further support for a loss of genetic diversity in this region of Australia during periods of extreme aridity.

## Systematics

### *Heteronotia* Wermuth, 1965 [62]

**Type species.** — *Heteronota binoei* Gray, 1845 – by monotypy.

**Diagnosis.** — A genus of moderately small (∼50 mm SVL), slender gekkonine lizards with small narrow head and long slender tail tapering to a point; rostral and mental shields rounded; labials much larger than neighboring scales, 4 enlarged postmentals, digits long and slender, with claw between three enlarged scales; no enlarged apical lamellae, single row of enlarged transverse lamellae beneath digits; precloacal pores present in males, cloacal spurs not greatly enlarged.

### Heteronotia spelea Kluge, 1963

Pilbara Cave Gecko

Figs 5a, 6a, 8–10

**Holotype.** — WAM R12638 (female), collected from Prophecy West mine, Bamboo Creek, WA, by A.M. Douglas and W.D.L. Ride on 12 or 13 October 1957.

**Paratypes.** — WAMR12639–40; collection details as for holotype.

**Diagnosis.** — Distinguished from congeners by medium body size (to 55.5 mm SVL), gracile habitus, elongate head (to 11.28 mm HeadW), long slender limbs and tail, small tympanum, typically 9 or 10 supralabials and 7 or 8 infralabials, enlarged dorsal tubercles surrounded by at least one smaller granule, dorsum with four or five strongly-contrasting dark bands from nape to hind limbs, and bands with straight edges.

**Description.** — Body size moderate (range 42.00–55.50 mm SVL); body slender, dorso-ventrally compressed in cross-section with flattened venter; head triangular, with moderately elongate snout with rounded tip; head slightly dorso-ventrally compressed; rostral rectangular, twice as broad as deep; dorsomedial rostral crease extending ventrally halfway from top of rostral; nostril surrounded by rostral, first supralabial, one postnasal and two supranasals; anterior supranasals greatly enlarged, in contact at midline; supralabials (9–10); mental triangular and broader than long; inner postmentals enlarged, twice as long as broad and in broad contact; outer postmentals ∼¼ size of inner postmentals, in point contact with first infralabial; remaining gular scales small, granular; infralabials (7–8); small ear opening.

Scales on top of head small and rounded, becoming keeled on nape; enlarged dorsal tubercles strongly keeled and surrounded by at least one small granule, in 12–16 (usually 12 or 14) longitudinal rows of enlarged, keeled dorsal tubercles at midbody; in males, precloacal pores 2–6, spurs not greatly enlarged.

Limbs and digits long and slender; finger length: 3>4>2>5>1; toe length: 4>3 = 5>2>1; top of limbs with slightly enlarged keeled scales, scales in contact (not separated by granules); ventral surface of hind limb and precloacal region with enlarged flattened scales. Tail long (to 72 mm) and thin, tapering to a fine point.

**Pattern and coloration.** — Dorsum with strongly contrasting alternating dark and pale bands; bands of similar width; dark bands 4; top of head light brown, often mottled; clearly-defined dark brown temporal stripe in contact with usually nuchal band; dark loreal stripe usually present, less defined than temporal stripe; dorsal surfaces of limbs light brown (unbanded); original tails with alternating banding, brown bands wider than pale bands, 8–9 dark bands on original tails; gular region moderately stippled, venter pale with light stippling, plantar and palmar surfaces dark.

**Habitat.** — Sheltering among rocks, especially in crevices, caves and mines.

**Distribution.** — The Pilbara bioregion. South-western outlying records occur at Uaroo Station, Barlee Range and also a single record from further south in the Kennedy Range.

**Etymology.** — *spelea* refers to this species' cave-dwelling habits.

### Heteronotia fasciolatus sp. nov. ZooBank LSID:

urn:lsid:zoobank.org:act:5BDF1C73-D8F8-4EB2-A737-E09966817610

Pale-headed Gecko, Figs. 5c, 6b, 11, 12

**Holotype.** — NTM R36284 (male), collected from Harts Range racecourse, central Australia, NT (−23.02284°S, 134.93135°E), on 3 June 2010 by M. Fujita, M. Pepper, and C. Moritz.

**Paratypes.** — MCZ 188177 Harts Range racecourse (−23.02284°S, 134.93135°E); MCZ 188183 (male) Tylers Pass, NT (−23.62938°S, 132.35660°E); MCZ 188182 (male), Ormiston Gorge, NT (−23.63799°S, 132.73291°E); NTM R36288 (female) Tylers Pass, NT (−23.62938°S, 132.35660°E); NTM R36297 (male), Ormiston Gorge, NT (−23.63799°S, 132.73291°E).

**Diagnosis.** — Distinguished from congeners by medium body size (to 57.0 mm SVL), slightly robust habitus, moderately wide head (to 13.7 mm HeadW), tail stout at base, small tympanum, typically 8 or 9 supralabials and 6 or 7 infralabials, enlarged dorsal tubercles surrounded by at least one smaller granule anterior and posterior to scale, and usually two smaller granules to sides, dorsum with 6–8 strongly contrasting bands; edges of bands with dark brown border and irregular edge (some bands breaking up), top of head pale.

**Description.** — Body size moderate (range 34.00–57.00 SVL); body stout, dorso-ventrally compressed in cross-section with flattened venter; head triangular, with short snout with broadly rounded tip; neck only slightly constricted; head slightly dorso-ventrally compressed; rostral rectangular, twice as broad as deep; dorsomedial rostral crease extending halfway from top of rostral; nostril surrounded by rostral, first supralabial, one postnasal and two supranasals; anterior supranasals greatly enlarged, in contact at midline; supralabials (7–10); mental triangular and broader than long; inner postmentals enlarged, twice as long as broad and in broad contact; outer postmentals ∼¼ size of inner postmentals, in point contact with first infralabial; remaining gular scales small, granular; infralabials (5–7); small ear opening.

Scales on top of head small and rounded, becoming keeled on nape, keeled scales on nape widely separated by granular scales; enlarged dorsal tubercles strongly keeled and surrounded by at least one small granule anterior and posterior to scale, and usually two (occasionally one) to either side of scale, in 14 longitudinal rows of enlarged, keeled dorsal tubercles at midbody; in males, precloacal pores 4, spurs not enlarged.

Limbs and digits long and slender; finger length: 3>4>2>5>1; toe length: 4>5>3>2>1; top of limbs with rounded weakly keeled scales; on arms, scales in contact (not separated by granules); on legs, scales slightly separated by granules; ventral surface of hind limb and precloacal region with enlarged flattened scales. Tail long (to 71 mm), stout at base then tapering to a fine point.

**Pattern and coloration.** — Pale head, suffused with light red or brown pigment; brown and pale contrasting bands on dorsum (5–8 brown bands), separated by dark brown border; bands without straight edges, sometimes breaking up or combining with other bands; narrow dark brown temporal and loreal stripes usually clearly-defined; similar-sized alternating pale and dark bands on tail (10–13 dark bands). Undersurfaces pale cream.

**Habitat.** — Sheltering among rocks naturally, but can be found among human-made structures such as sheet metal in rocky surrounds.

**Distribution.** — Known from the east and west Macdonnell Ranges (and including Harts Range) of central Australia. Not occurring north of Harts Range or at Mt. Doreen, or south in the Gardiner and James Ranges.

**Etymology.** — In a PhD thesis, Stewart (1996) informally proposed the name *H. fasciolatus* based on four karyotyped specimens from Undoolya Station in Central Australia. *Fasciolatus* is a diminutive of the Latin *fascia*, meaning ‘band’.

**Remarks.** — This species occurs in sympatry with the CA6 chromosome race of *H. binoei* in central Australia. Here *H. binoei* also posess a banded morphology, with occasional samples strikingly similar to *H. fasciolatus* **sp. nov**.

**Comparison to other species.** — *Heteronotia fasciolatus* **sp. nov**. can be distinguished from *H. spelea* and *H. atra* **sp. nov**. by the strongly contrasting dorsal bands being uneven and often broken, and never straight edged. Furthermore, the head is broad and pale, with light red or brown pigment. The enlarged dorsal tubercles are widely spaced compared to *H. spelea* and *H. atra* **sp. nov**., generally with two small granular scales separating them. It can be further differentiated from *H. atra* **sp. nov.** by having a small ear opening.

### Heteronotia atra sp. nov

ZooBank LSID urn:lsid:zoobank.org:act:DC8FEABC-1DF0-4EED-A340-EF6ABF4A76C3

Black Pilbara Gecko, Figs. 5b 6c, 13, 14

**Holotype.** — WAM R110076 (male) collected 5 km south of Lake Poongkaliyarra, WA (−21.03590°S, 117.10658°E) on 10 October 2004 by J.K. Rolfe, L.A. Smith, and B. Durrant

**Paratypes.** — WAM R110056 (female), WAM R165222 (female), WAM R162439 (female) WAM R110075 (female), same location as holotype; WAM R163243 (male), 12.8 km southwest of Roebourne, WA (−20.8877°S, 117.1017°E).

**Diagnosis.** — Distinguished from congeners by large body size (to 62.5 mm SVL), gracile habitus, elongate head (to 11.72 mm HeadW), long slender limbs and tail, greatly enlarged tympanum, typically 10 supralabials and 8 infralabials, enlarged dorsal tubercles in contact with each other at anterior and posterior edges of scale and usually to either side or separated with at most a few small granules, and melanistic ‘charcoal’ coloration.

**Description.** — Body size large (range 54.00–62.5 mm SVL); body slender, dorso-ventrally compressed in cross-section with flattened venter; head triangular, with elongate snout with broadly rounded tip; neck moderately constricted; head slightly dorso-ventrally compressed; rostral rectangular, twice as broad as deep; dorsomedial rostral crease extending halfway from top of rostral; nostril surrounded by rostral, first supralabial, one postnasal and two supranasals; anterior supranasals greatly enlarged, in contact at midline; supralabials (8–10); mental triangular and broader than long; inner postmentals enlarged, twice as long as broad and in broad contact; outer postmentals ∼¼ size of inner postmentals, in point contact with first infralabial; remaining gular scales small, granular; infralabials (7–8); large ear opening.

Scales on top of head small and rounded, becoming keeled on nape, keeled scales largely in contact, separated by few granular scales; enlarged dorsal tubercles strongly keeled and in contact with adjacent scales at anterior and posterior edges, and usually also with adjacent scales to either side but with at most a single granule; vertebral zone with a hiatus of enlarged tubercles and 3–5 granules, in 14–18 (usually 16) longitudinal rows of enlarged, keeled dorsal tubercles at midbody; in males, precloacal pores 4–7, spurs not enlarged.

Limbs and digits long and slender; finger length: 3>4>2>5>1; toe length: 4>3>5>2>1; top of limbs with rounded weakly keeled scales; on arms and legs, scales in contact (not separated by granules); ventral surface of hind limb and precloacal region with enlarged flattened scales. Tail long and slender (to 74 mm), tapering to a fine point.

**Pattern and coloration.** — In life, a uniform charcoal or black-brown ground color usually with no indication of bands; dark brown temporal stripe present, terminating above tympanum; loreal stripe usually present, less defined than temporal stripe; gular region moderately stippled, venter pale cream with light stippling, plantar and palmar surfaces dark. In preservative, dorsal surface uniform dark brown, occasionally with alternating bands just discernible in juveniles; vertebral zone dark brown, sometimes unpigmented on anterior portion dorsum.

**Habitat.** — Bare black boulder scree (Cooya Pooya dolerite) with scattered *Triodia* and shrubs.

**Distribution.** — This species is known from a geologically distinctive basaltic, flat-topped mesa near Karratha in the northwest Pilbara region. Specimen WAM R163253 was collected nearby but not on the mesa itself.

**Etymology.** — The specific name *atra* (Latin) means ‘black’, and refers to the melanic coloration.

**Comparisons with other species.** — *Heteronotia atra* **sp. nov**. can be distinguished from *H. spelea* and *H. fasciolatus* **sp. nov.** by the melanic ‘charcoal’ colouration and greatly enlarged external ear opening. The enlarged dorsal tubercles are almost in contact, with few small granular scales in between, in contrast to *H. spelea* and *H. fasciolatus* **sp. nov**. where the enlarged tubercles are spaced more widely apart.

## Supporting Information

Table S1 **Summaries of morphological characters measured.** Body proportion variables were used in the multivariate analyses.
(XLS)
